# Illinois Veterinary and Surgical Association

**Published:** 1901-03

**Authors:** W. A. Swain

**Affiliations:** Secretary


### ILLINOIS VETERINARY AND SURGICAL ASSOCIATION.

THE twelfth annual meeting was held at Decatur on Wednesday, January 9, 1901, with President V. G. Hunt in the chair. The roll-call was responded to by a goodly number. The minutes of the previous meeting were read and approved.

On motion, the chair appointed a committee of three to draft resolutions indorsing the candidacy of Dr. W. J. Martin, of Kankakee, for the office of State veterinarian. A copy of the resolutions was signed by the President and Secretary and forwarded to Governor Yates at Springfield.

Dr. V. G. Hunt, being first on the programme for a paper on "Metritis," gave a very interesting and instructive account of this disease and the various modes of treatment, and was responded to by Dr. S. H. Swain and Dr. S. D. Brown. Dr. W. A. Swain next read a paper on "Muscular Rheumatism;" responded to by Drs. L. C. Pray, V. G. Hunt, and C. A. Hurlbutt. Dr. A. Travis then delivered a short talk on the "Treatment of Stomatitis;" responded to by Dr. H. W. Leavitt. An hour was then devoted to the discussion of the foregoing subjects, after which adjournment was taken for dinner.

Reports of cases being taken in order, many interesting subjects were introduced and discussed at length.

At 10.30 P.M. the entire Association partook of an oyster supper at the expense of Neisler & Burwell, wholesale druggists, and adjournment was taken until 8 A.M., January 10th.

*January 10th.* Meeting called to order by President Hunt. Dr. R. W. Brathwait being absent, Dr. C. A. Hurlbutt gave the Association an interesting talk on "Antiseptic Surgery;" responded to by Dr. S. D. Brown. Dr. S. H. Swain, being next on the programme, read a very concise essay on "Equine Canker;" responded to by Drs. V. G. Hunt and W. J. Martin.

On motion, the length of time allowed to each person having the floor was limited to three minutes.

On motion, the location and date of the next meeting were fixed at Decatur, Ill., on August 8 and 9, 1901.

The Association next proceeded to the election of officers for the ensuing year: Dr. V. G. Hunt, of Arcola, was re-elected by acclamation to fill the office of President. First Vice-President, Dr. John Osborne, of Nokomis, Ill.; Second Vice-President, Dr. D. K. Goodale, of Mt. Vernon, Ill.; Secretary, Dr. W. A. Swain, of Mt. Pulaski, Ill.; Treasurer, Dr. S. H. Swain, of Decatur, Ill.

Standing Committees appointed were: Committee on Programme—Drs. V. G. Hunt, S. H. Swain, and W. A. Swain. Committee on Membership—Drs. S. H. Swain, John Osborne, J. R. Groves, J. M. Reed, and W. C. Dawson. Committee on Legislation—Drs. V. G. Hunt, S. H. Swain, and John Osborne.

The meeting then adjourned until August 8 and 9, 1901.

W. A. Swain,
Secretary.

